# Design and synthesis of ERα agonists: Effectively reduce lipid accumulation

**DOI:** 10.3389/fchem.2022.1104249

**Published:** 2022-12-08

**Authors:** Jinfei Yang, Weiwei Yao, Huihui Yang, Yajing Shen, Yuanyuan Zhang

**Affiliations:** ^1^ School of Health and Life Sciences, University of Health and Rehabilitation Sciences, Qingdao, China; ^2^ West China School of Pharmacy, Sichuan University, Chengdu, China

**Keywords:** ERα, agonists, lipid accumulation, NAFLD, liver disease

## Abstract

In recent years, the incidence of non-alcoholic fatty liver disease (NAFLD) has been increasing worldwide. Hepatic lipid deposition is a major feature of NAFLD, and insulin resistance is one of the most important causes of lipid deposition. Insulin resistance results in the disruption of lipid metabolism homeostasis characterized by increased lipogenesis and decreased lipolysis. Estrogen receptor *α* (ERα) has been widely reported to be closely related to lipid metabolism. Activating ERa may be a promising strategy to improve lipid metabolism. Here, we used computer-aided drug design technology to discover a highly active compound, YRL-03, which can effectively reduce lipid accumulation. Cellular experimental results showed that YRL-03 could effectively reduce lipid accumulation by targeting ERα, thereby achieving alleviation of insulin resistance. We believe this study provides meaningful guidance for future molecular development of drugs to prevent and treat NAFLD.

## 1 Introduction

**FIGURE 1:**
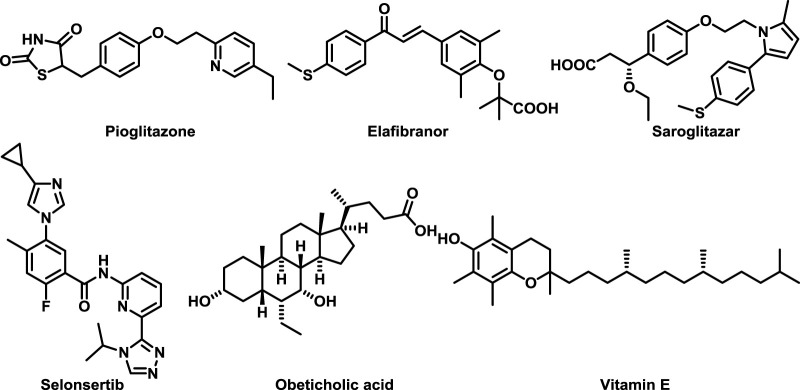
Drug molecules that have entered clinical phase III.

Non-alcoholic fatty liver disease (NAFLD) has become the most common chronic liver disease in the world (Li et al., 2018), affecting more than 30% of the general population in western countries, and its incidence continues to increase in other parts of the world (Asrih and Jornayvaz, 2015). NAFLD is a multifactorial disease triggered by interactions between environment, genetic background, and metabolic stress (Lonardo et al., 2017). Unlike alcoholic fatty liver disease, patients have no history of excessive alcohol consumption (Ahmed, 2015). The pathogenic mechanisms involved in NAFLD are complex and have not yet been fully elucidated (Xi and Li, 2020; Hrncir et al., 2021). Insulin resistance is one of the key factors in the development of steatosis (Alam et al., 2016), which leads to an imbalance between hepatic lipogenesis and metabolism, mainly manifested by increased *de novo* lipogenesis and decreased adipose tissue lipolysis (Saponaro et al., 2015). If NAFLD is not controlled, it will further develop into liver cirrhosis (Zhou et al., 2020), and may eventually develop into hepatocellular carcinoma, which seriously threatens human health (Chrysavgis et al., 2022). However, there is no drug specifically for the treatment of NAFLD on the market so far (Ma et al., 2019; Wang et al., 2020). Some drugs that regulate metabolism, oxidative stress and anti-fibrosis are still in the clinical stage (Kumar et al., 2021), such as Pioglitazone (Della Pepa et al., 2021), Elafibranor (Boeckmans et al., 2022), Saroglitazar (Gawrieh et al., 2021), Obeticholic acid (Abenavoli et al., 2018; Malnick et al., 2020), Selonsertib (Reimer et al., 2020) and Vitamin E (Perumpail et al., 2018) (Figure 1). Therefore, there is an urgent need to develop novel drugs with high efficacy and minimal side effects for the treatment of NAFLD. Lipid metabolism plays a key role in the progression of NAFLD (Lai et al., 2016), and insulin resistance is one of the most important causes of lipid deposition. Insulin resistance results in the disruption of lipid metabolism homeostasis characterized by increased lipogenesis and decreased lipolysis (Yao et al., 2016). Estrogen receptor *α* (ERα) has been widely reported to be closely related to lipid metabolism, especially has an important impact on NAFLD (Meda et al., 2022). Studies have shown that the activation of ERα can effectively reduce the accumulation of liver lipids (Chen et al., 2020). Based on this, we plan to use computer-assisted drug design technology to develop an agonist of ERα, which can effectively reduce lipid accumulation by targeting ERα, thereby achieving the purpose of alleviating NAFLD.

## 2 Results and discussion

**FIGURE 2:**
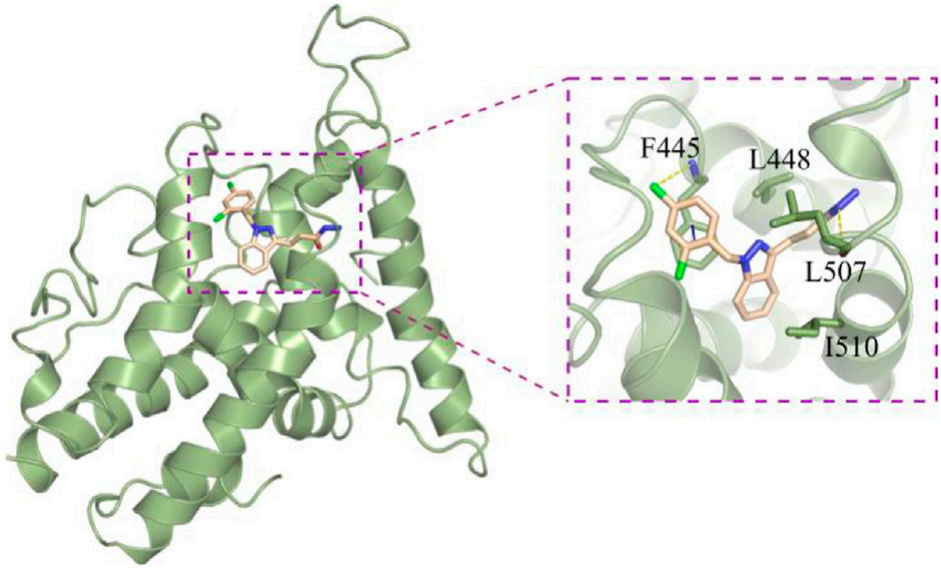
Computer-assisted molecular docking experiments (PDB: 5GS4).

For the target of ERα, we used computer-aided drug design technology to discover a compound 3-(1-(2,4-dichlorobenzyl)-1H-indazol-3-yl)propanehydrazide (YRL-03) with strong interaction with ERα (Figure 2). Molecular docking experiments show that the chlorine atom on the benzene ring of YRL-03 has a strong electrophilic interaction with the amino group on the amino acid site F445 of ERα, and the nitrogen atom on the hydrazide of YRL-03 has a strong electrophilic interaction with the carbonyl group on the amino acid site L507. In addition, the benzene ring of YRL-03 has an arene-arene interaction with the benzene ring of F445.

**SCHEME 1:**
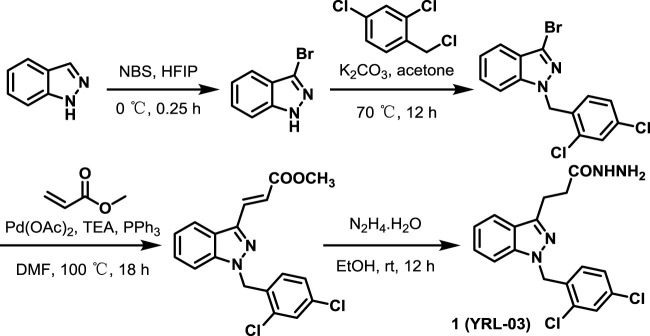
Chemical synthesis of YRL-03^*a*^.

^*a*^Reaction conditions: (a) NBS, Hexafluoroisopropanol (HFIP), 0°C, 0.25 h; (b) 2,4-dichloro-1-(chloromethyl)benzene, K_2_CO_3_, acetone, 70°C, 12 h, 78% (two steps); (c) methyl acrylate, Pd(OAc)_2_, TEA, PPh_3_, DMF, 100°C, 18 h, 81%; (d)N_2_H_4_. H_2_O, EtOH, rt, 12 h, 96%.

Subsequently, starting from cheap and easily available indazole, we used the developed chemical synthesis route to rapidly synthesize the target molecule YRL-03 by a four-step reaction, and the total yield of four-step reaction is 43.6% (Scheme 1). To our delight, the whole reaction process is mild, no nitrogen protection is required, and no hazardous reagents are used.

With the obtaining target molecule YRL-03 in hand, we began to try to verify whether this compound has the effect of reducing lipid accumulation. The cellular model is a model of lipid deposition in hepatocytes induced by oleic acid. First, hepatocytes were treated with 125 μm sodium oleate to induce lipid deposition, resulting in a uniform distribution of lipid droplets in hepatocytes without significant changes in cell morphology. Second, YRL-03 was formulated into five gradient concentrations of 6.25, 12.5, 25, 50, and 100 μm, respectively, to verify the effect of different gradient concentrations on lipid deposition. Cell experiments showed that lipid droplets were significantly reduced at 6.25 μm, but little change at 12.5, 25, 50, and 100 μm. It reveals that YRL-03 had a good effect of reducing lipid accumulation under the administration of 6.25 μm gradient concentration.

**FIGURE 3:**
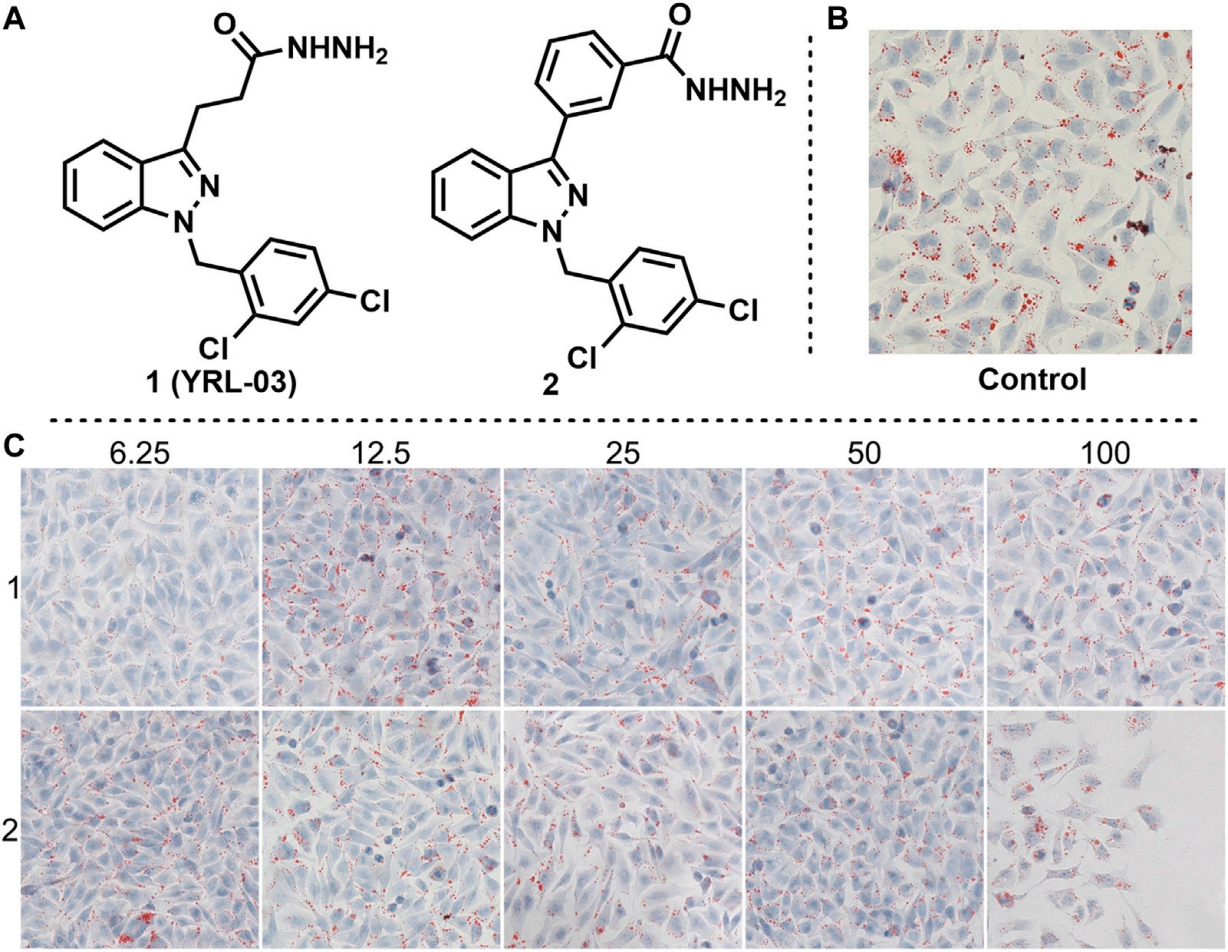
Effects of YRL-03 and compound 2 on lipid accumulation. **(A)** Chemical structure of YRL-03 and compound 2. **(B)** Control experiment. **(C)** Effects of different concentration gradients of YRL-03 and compound 2 on lipid accumulation.

In order to find more excellent active compunds, we imagined that if the 3-position alkyl hydrazide of the indazole was changed to an aryl hydrazide, would it produce better results? Based on this hypothesis, we synthesized compound 2 by a similar synthetic method, and tested its effect on lipid accumulation. Regrettably, the test results showed that compound 2 did not show a good effect on reducing lipid accumulation. When the gradient concentration was 6.25 μm, lipid deposition did not decrease but increased. When the gradient concentration increased to 100 μm, the cell morphology changed significantly (Figure 3).

**FIGURE 4:**
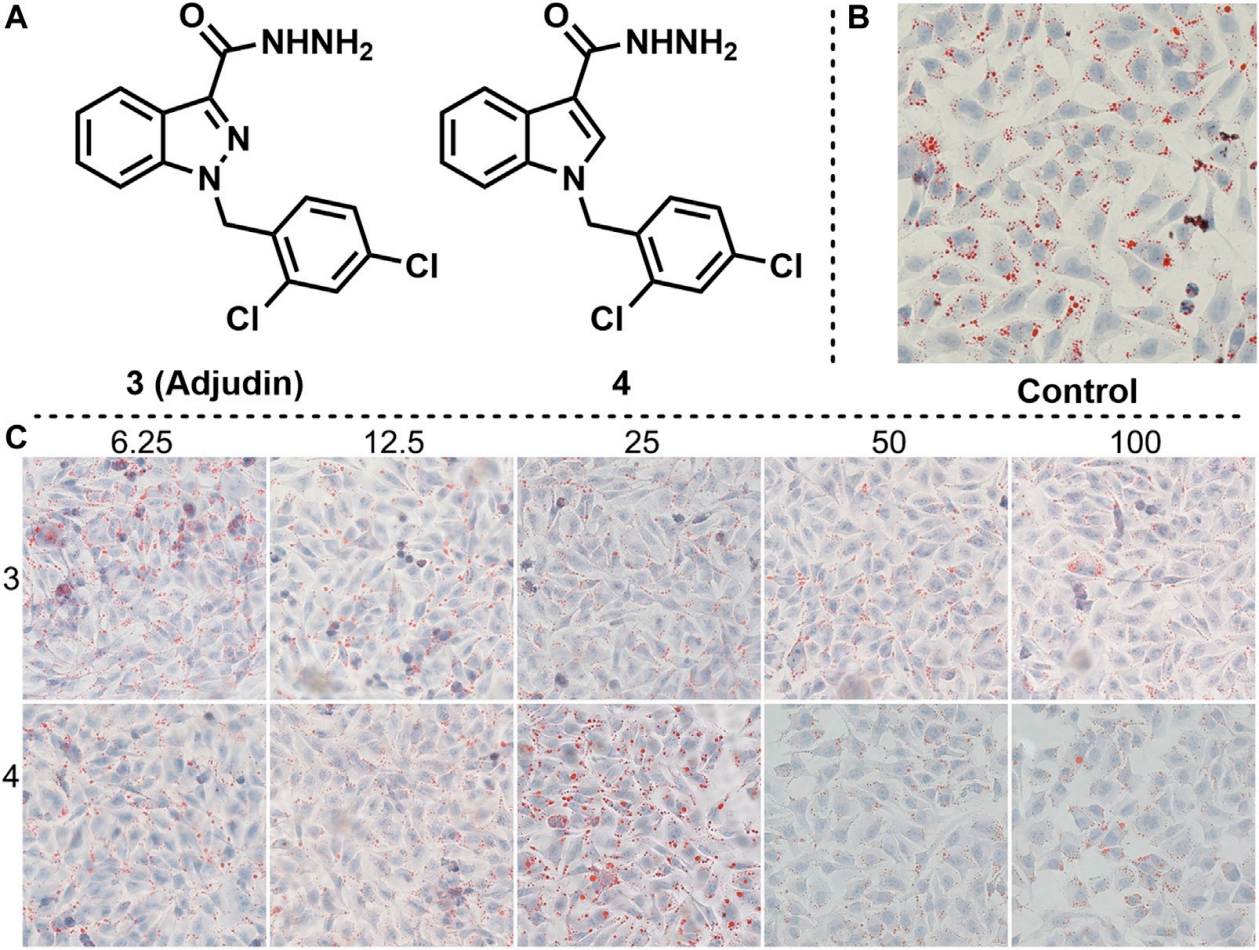
Effects of compounds 3 and 4 on lipid accumulation. **(A)** Chemical structure of compounds 3 and 4. **(B)** Control experiment. **(C)** Effects of different concentration gradients of compounds 3 and 4 on lipid accumulation.

Adjudin is a potential non-hormonal male contraceptive under development (Mruk et al., 2006). We found that adjudin and its derivatives also have interaction with ER in previous studies, but the cell experiments showed that they had little inhibitory effect on estrogen receptors (Yao et al., 2022). Therefore, we envisioned whether they would have the opposite effect, being an estrogen receptor agonist. Based on this idea, we synthesized Adjudin and its derivative 4, and verified their effects on lipid deposition at different gradient concentrations (Figure 4, the same control used for compounds 1, 2, 3 and 4). The results of cell experiments showed that Adjudin did not reduce lipid deposition at low concentrations. When the concentration was increased to 50 and 100 μm, it had a weak effect on reducing lipid deposition. The low concentration of compound 4 did not reduce lipid deposition, and when the concentration was increased to 50 and 100 μm, lipid droplets were reduced, but cell morphology was changed.

**FIGURE 5:**
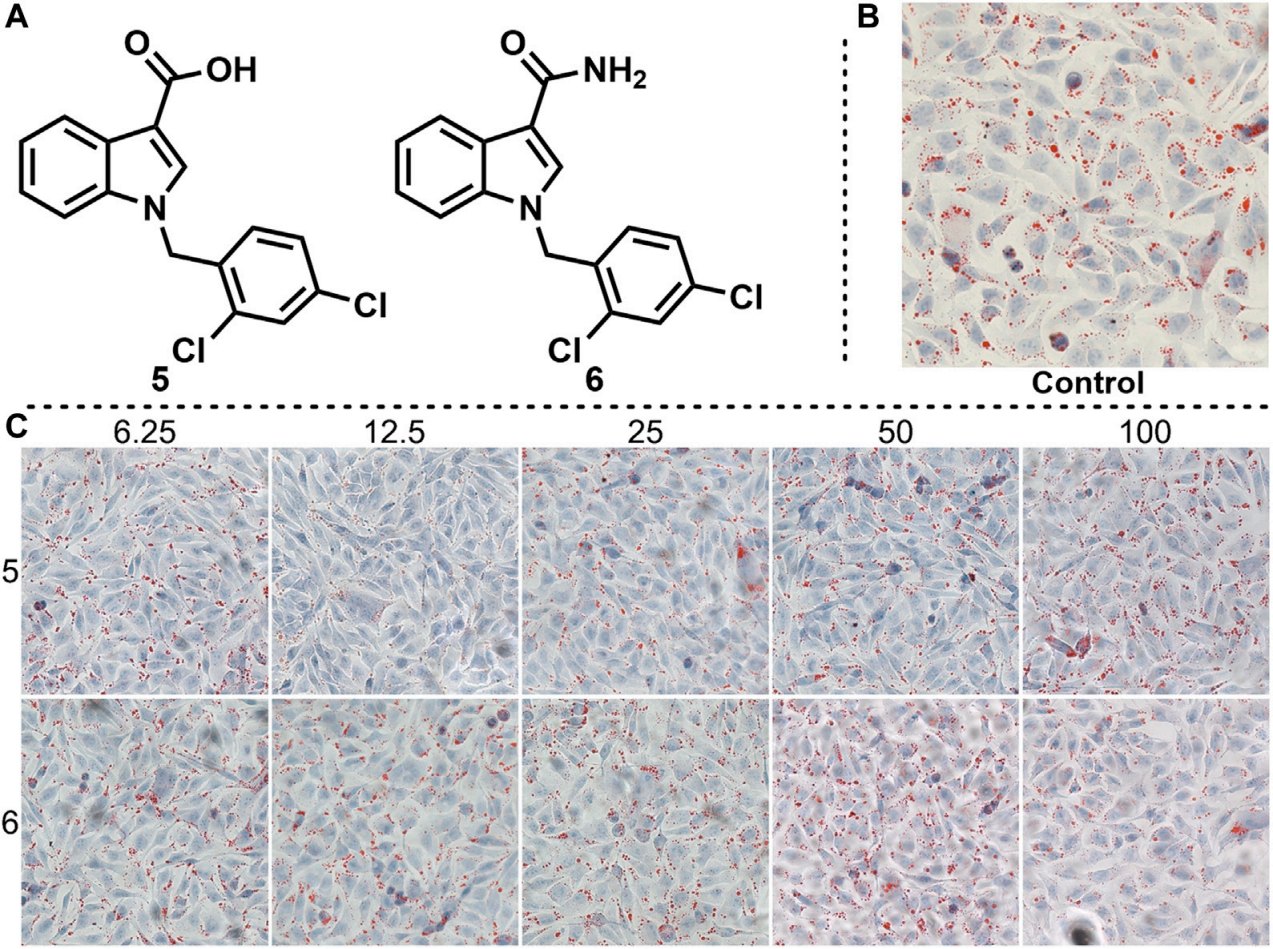
Effects of compounds 5 and 6 on lipid accumulation. **(A)** Chemical structure of compounds 5 and 6. **(B)** Control experiment. **(C)** Effects of different concentration gradients of compounds 5 and 6 on lipid accumulation.

Given that compound 4 has a certain lipid-lowering effect at 50 and 100 μm, we imagined whether changing the hydrazide substituent on the indole backbone of compound 4 to carboxyl or amide could enhance its lipid-lowering effect. Based on this idea, we synthesized compounds 5 and 6, and tested their effects on fat accumulation (Figure 5). The cell experiments results showed that the two compounds had no effect on reducing fat accumulation. Even with increasing their concentration, lipid droplets are still present in abundance.

**FIGURE 6:**
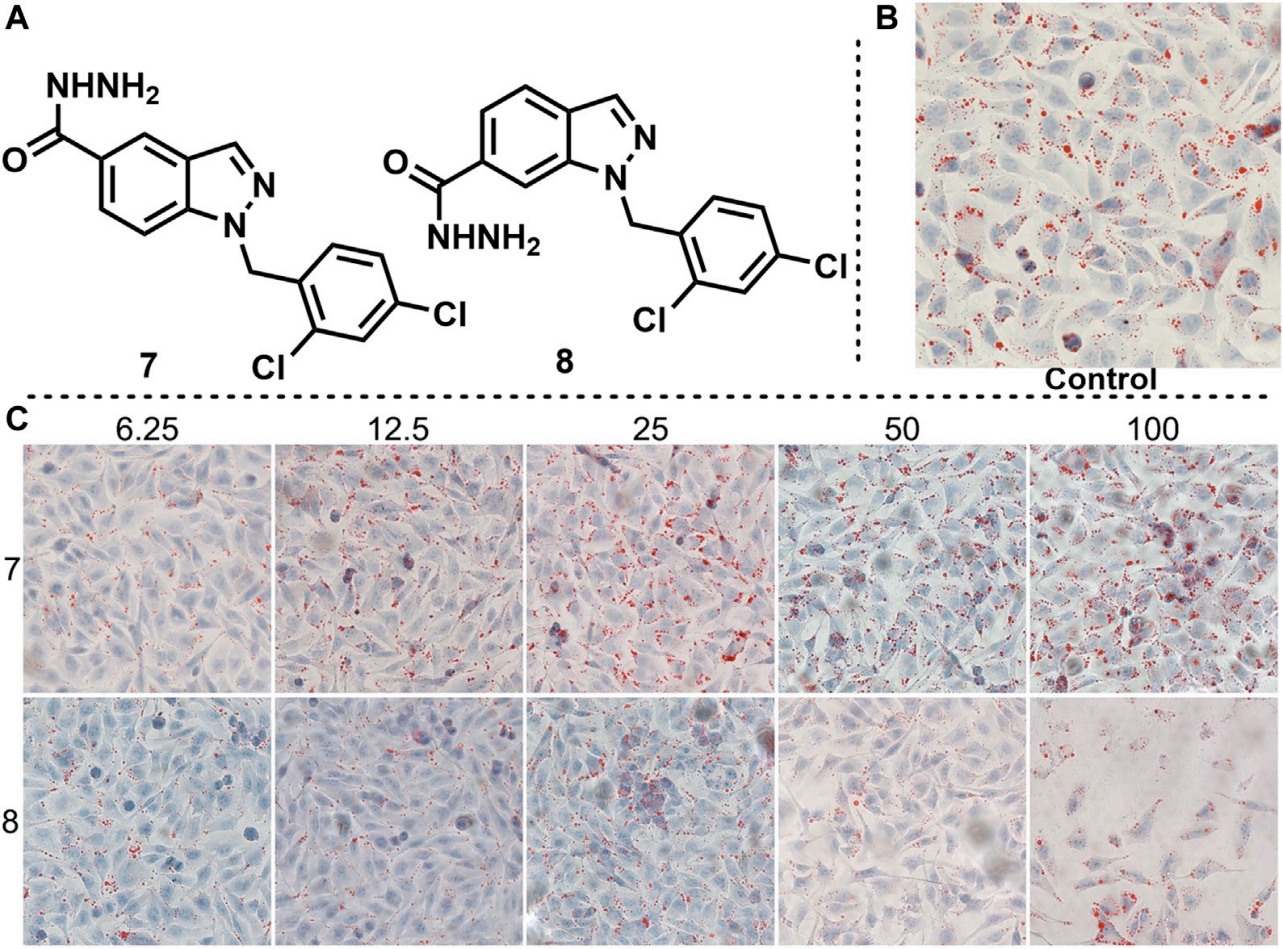
Effects of compounds 7 and 8 on lipid accumulation. **(A)** Chemical structure of compounds 7 and 8. **(B)** Control experiment. **(C)** Effects of different concentration gradients of compounds 7 and 8 on lipid accumulation.

The position of the substituent has a strong correlation with the biological activity of the compound. Therefore, the biological activity may also change greatly when the position of the substituent changes. To improve the biological activity of ER-targeted agonists, we changed the hydrazide position on the adjudin indazole backbone from 3 to 5 or 6 to obtain compounds 7 and 8, and tested their effects on fat accumulation (Figure 6, the same control used for compounds 5, 6, 7 and 8), respectively. Unfortunately, it didn’t end up as we expected. Cell experiments showed that compounds 7 and 8 did not reduce fat accumulation. When the gradient concentration of compound 8 was increased to 50 and 100 μM, the cell morphology changed significantly.

## 3 Conclusion

In conclusion, we developed an ERα-targeting agonist YRL-03 by computer-aided drug design technology, which was effective in reducing lipid accumulation at a concentration of 6.25 μm. Cell experiments showed that YRL-03 could effectively inhibit lipid accumulation. The specific interaction mode of YRL-03 and ERα given by molecular docking experiments is that the chlorine atom on the benzene ring of YRL-03 has a strong electrophilic interaction with the amino group on the amino acid site F445 of ERα, and the benzene ring of YRL-03 have aromatic-aromatic interactions with the benzene ring of F445, the nitrogen atom on the YRL-03 hydrazide has a strong electrophilic interaction with the carbonyl group on the amino acid site L507. Notably, the target compound can be obtained from cheap and easily available indazole through four-step reactions, and the whole reaction process is simple to operate and does not need flammable and explosive dangerous reagents. Further molecular structure optimization and toxicology experiments are ongoing in our group. We believe that the results of this study will provide meaningful guidance for the future development of drugs that can effectively treat NAFLD.

## 4 Experimental section

General Information Unless stated otherwise, all reactions were conducted in pressure tubes under N_2_. All solvents were received from commercial sources without further purification. Commercially available reagents were used as received. Non-commercially available substrates were synthesized following reported protocols. Thin-layer chromatography (TLC) was visualized using a combination of UV and potassium permanganate staining techniques. Silica gel (particle size 40–63 μm) was used for flash column chromatography. NMR spectra were recorded on Bruker AV 400 spectrometer at 400 MHz (^1^H NMR), 100 MHz (^13^C NMR). Proton and carbon chemical shifts are reported relative to the solvent used as an internal reference. The results of molecular docking experiments were completed using Schrödinger and Molecular Operating Environment (MOE).

Typical Procedure for Synthesis of Compund 1. Indazole (5 mmol) was added to a stirred mixture of NBS (5.5 mmol, 1.1 equiv.) in HFIP (15 ml). After 0.25 h at 0°C, the organic layer was washed successively with aq NaHCO_3_, and brine, dried over anhydrous sodium sulphate, filtered, and evaporated *in vacuo*. Then the crude material was dissolved in acetone, and to the mixture 2,4-dichloro-1-(chloromethyl)benzene (5.5 mmol, 1.1 equiv.), K_2_CO_3_ (22 mmol, 4.4 equiv.) were added. The reaction mixture was refluxed overnight at 70°C. Then it was cooled to room temperature, filtered, and the residue was washed with acetone. The combined filtrate was concentrated under vacuum. The solid was dissolved in DCM and filtered to remove any undissolved solid. The residue was re-crystallized (DCM/hexane) to afford the pure product 3-bromo-1-(2,4-dichlorobenzyl)-1H-indazole as a white solid in 78% yield (two steps).

To a glass pressure tube were added 3-bromo-1-(2,4-dichlorobenzyl)-1H-indazole (0.5 mmol, 1.0 equiv.), Pd (OAc)_2_ (0.05 mmol, 10 mol%), PPh_3_ (0.1 mmol, 20 mol%) and anhydrous DMF (2 ml) under N_2_. and then TEA (1.5 mmol, 3.0 equiv.) and methyl acrylate (5 mmol, 10.0 equiv.) were added. The resulting solution was stirred at 100°C for 18 h. Cool the reaction mixture and dilute with EA. Wash with waterand dry over Na_2_SO_4_. Evaporate and purify the residue by column chromatography to obtain the product methyl (E)-3-(1-(2,4-dichlorobenzyl)-1H-indazol-3-yl)acrylate in 81% yield.

To a solution of methyl (E)-3-(1-(2,4-dichlorobenzyl)-1H-indazol-3-yl)acrylate (1 mmol, 1.0 equiv.) in ethanol at room temperature was added hydrazine hydrate (50 mmol, 50.0 equiv.). The reaction mixture was stirred at room temperature over night. The volatiles were removed under reduced pressure and the crude mass was diluted with dichloromethane, washed with water, brine, dried over anhydrous sodium sulphate and the solvent was removed under reduced pressure to obtain the crude product. The residue was purified by column chromatography to afford the pure product 1 (YRL-03) as a white solid in 96% yield.

Cell Culture. Human hepatic L02 cells were obtained from the American Type Culture Collection, and cultured with Roswell Park Memorial Institute (RPMI) 1,640 medium (Hyclone, UT, United States) supplemented with 10% fetal bovine serum (Biological Industries, CT, United States) and 1% penicillin/streptomycin (Hyclone, UT, United States).

Oil red O staining. L02 cells were seeded in 24-well plates and induced with sodium oleate at 100 μm for 24 h when reaching 50% confluence. Then cells were treated with compounds at 6.25, 12.5, 25, 50, 100 μm for 24 h. Then cells were washed with phosphate buffer saline (PBS) and fixed with 4% paraformaldehyde (PFA, Sangon, Shanghai, China) for 20 min at room temperature, stained with freshly diluted oil red O staining solution (3 mg/ml) for 45 min, rinsed with PBS, and sealed with glycerin (Chu et al., 2019). Lipid droplets in cells were captured by an optical microscope (Zeiss).

3-(1-(2,4-dichlorobenzyl)-1H-indazol-3-yl)propanehydrazide (1). White solid (61% yield, four steps). ^1^H NMR (400 MHz, CDCl_3_) *δ* 7.72 (d, *J* = 8.0 Hz, 1 H), 7.42 (d, *J* = 2.0 Hz, 1 H), 7.40-7.35 (m, 1 H), 7.30-7.28 (m, 1 H), 7.18-7.14 (m, 2 H), 7.09 (dd, *J* = 8.0, 1.6 Hz, 1 H), 6.60 (d, *J* = 8.4 Hz, 1 H), 5.59 (s, 2 H), 3.83 (s, 2 H), 3.34 (t, *J* = 7.2 Hz, 2 H), 2.74 (t, *J* = 7.2 Hz, 2 H).^13^C NMR (100 MHz, CDCl_3_) *δ* 173.3, 144.8, 140.8, 134.0, 133.4, 133.0, 129.3, 129.3, 127.5, 127.1, 122.9, 120.6, 120.4, 109.1, 49.3, 33.0, 22.6.

3-(1-(2,4-dichlorobenzyl)-1H-indazol-3-yl)benzohydrazide (2).^[23]^ White solid (77% yield, two steps). ^1^H NMR (400 MHz, CDCl_3_) *δ* 8.35 (t, *J* = 1.6 Hz, 1 H), 8.15 (dt, *J* = 7.6, 1.2 Hz, 1 H), 8.06 (d, *J* = 8.0 Hz, 1 H), 7.78 (dt, *J* = 8.0, 1.2 Hz, 1 H), 7.59 (t, *J* = 7.6 Hz, 1 H), 7.49 (s, 1 H), 7.44 (d, *J* = 2.4 Hz, 1 H), 7.42-7.37 (m, 2 H), 7.29-7.27 (m, 1 H), 7.09 (dd, *J* = 8.4, 2.4 Hz, 1 H), 6.73-6.71 (m, 1 H), 5.72 (s, 2 H), 4.14 (s, 2 H).^13^C NMR (100 MHz, DMSO)*δ* 165.9, 142.9, 141.4, 134.2, 133.9, 133.3, 133.21, 133.16, 130.7, 129.6, 129.2, 129.1, 127.8, 127.0, 126.6, 125.5, 121.9, 121.2, 120.9, 110.3, 49.3.

## Data Availability

The original contributions presented in the study are included in the article/Supplementary Material, further inquiries can be directed to the corresponding authors.
