# Supplementary material for: Design and synthesis of ERα agonists: Effectively reduce lipid accumulation
Source: Front Chem. 2022 Dec 8;10:1104249. doi: 10.3389/fchem.2022.1104249 (PMC9772986; doi:10.3389/fchem.2022.1104249)

**Design and Synthesis of ERα Agonists: Effectively Reduce Lipid Accumulation**

Jinfei Yang,*^[a]^ Weiwei Yao,^[a]^ Huihui Yang,^[a]^ Yajing Shen,^[a]^ and Yuanyuan Zhang*^[b]^

^[a]^ School of Health and Life Sciences, University of Health and Rehabilitation Sciences, Qingdao 266001 (China)

^[b]^ West China School of Pharmacy, Sichuan University, Chengdu 610041 (China)

# NMR Spectra


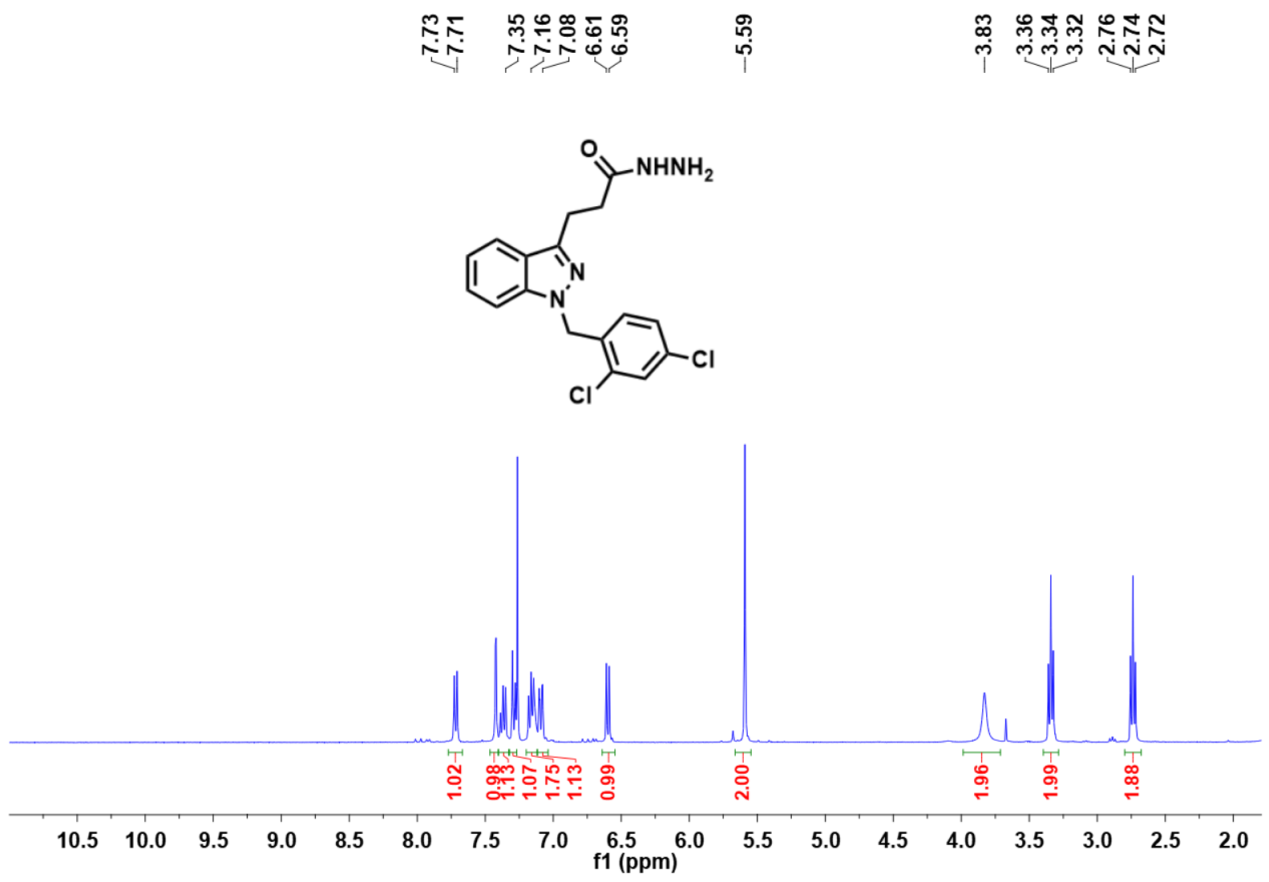


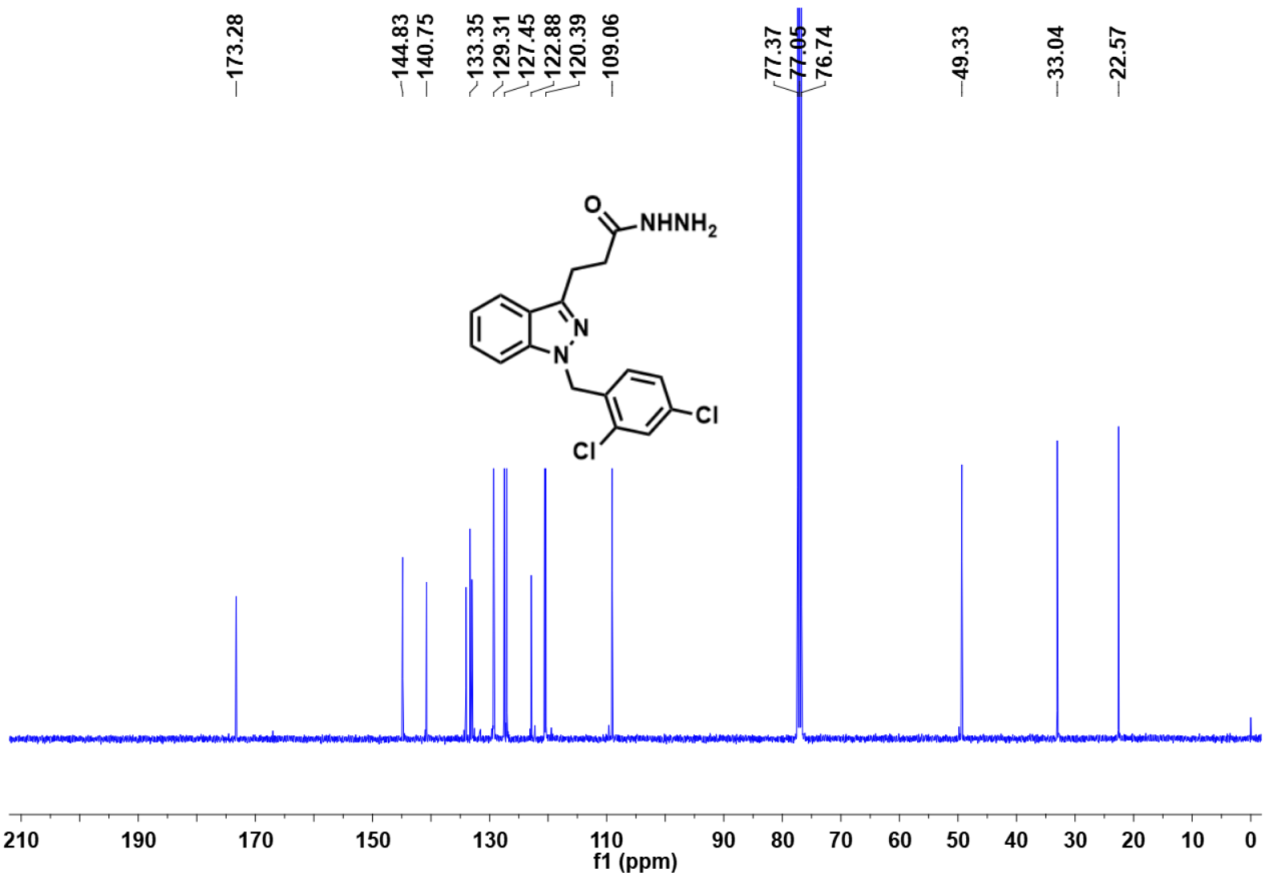


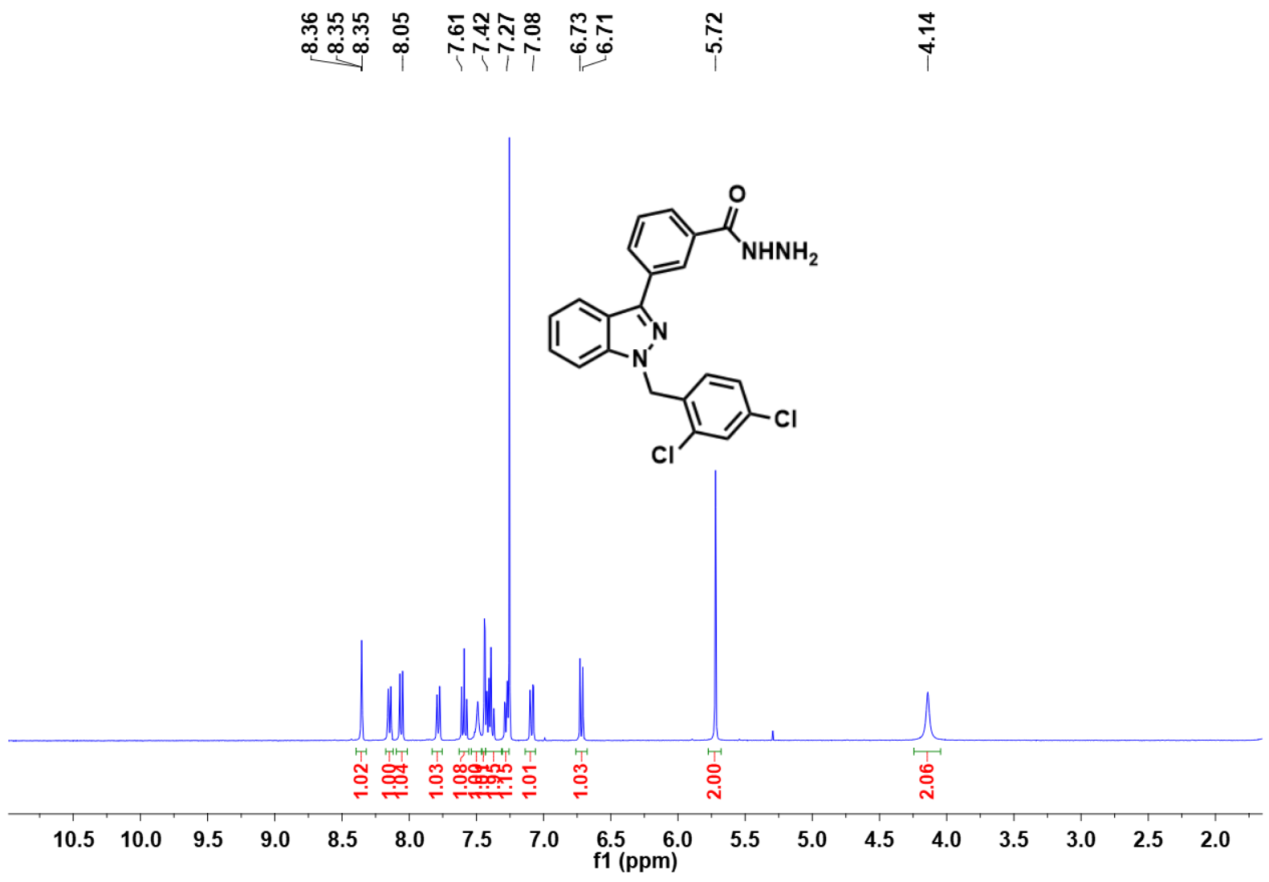


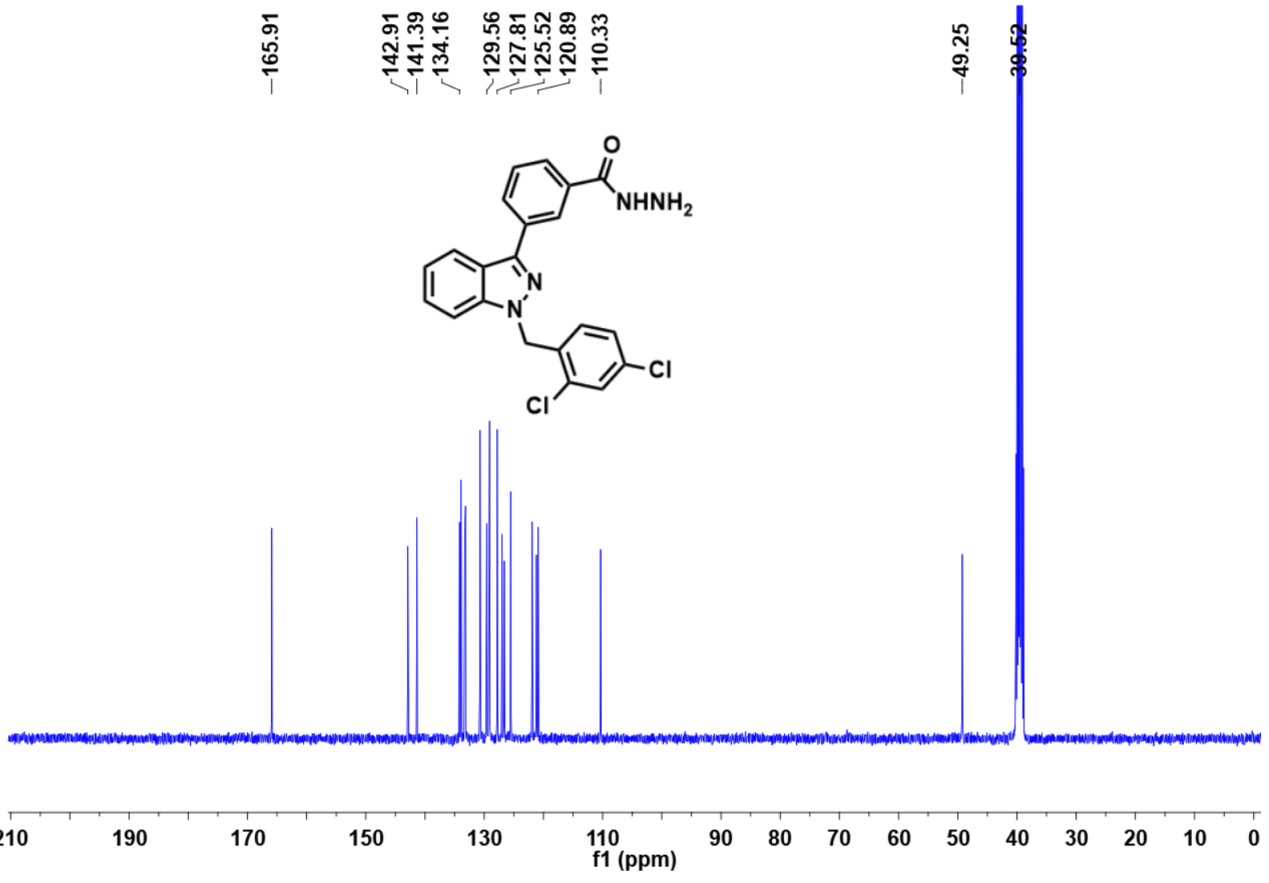

Supplement: Supplementary file 1 [file DataSheet1.docx]
